# A Quantitative Assessment Method for *Ascaris* Eggs on Hands

**DOI:** 10.1371/journal.pone.0096731

**Published:** 2014-05-06

**Authors:** Aurelie Jeandron, Jeroen H. J. Ensink, Stig M. Thamsborg, Anders Dalsgaard, Mita E. Sengupta

**Affiliations:** 1 Department of Infectious and Tropical Diseases, London School of Hygiene and Tropical Medicine, London, United Kingdom; 2 Department of Veterinary Disease Biology, Faculty of Health and Medical Sciences, University of Copenhagen, Copenhagen, Denmark; Queensland Institute of Medical Research, Australia

## Abstract

The importance of hands in the transmission of soil transmitted helminths, especially *Ascaris* and *Trichuris* infections, is under-researched. This is partly because of the absence of a reliable method to quantify the number of eggs on hands. Therefore, the aim of this study was to develop a method to assess the number of *Ascaris* eggs on hands and determine the egg recovery rate of the method. Under laboratory conditions, hands were seeded with a known number of *Ascaris* eggs, air dried and washed in a plastic bag retaining the washing water, in order to determine recovery rates of eggs for four different detergents (cationic [benzethonium chloride 0.1% and cetylpyridinium chloride CPC 0.1%], anionic [7X 1% - quadrafos, glycol ether, and dioctyl sulfoccinate sodium salt] and non-ionic [Tween80 0.1% -polyethylene glycol sorbitan monooleate]) and two egg detection methods (McMaster technique and FLOTAC). A modified concentration McMaster technique showed the highest egg recovery rate from bags. Two of the four diluted detergents (benzethonium chloride 0.1% and 7X 1%) also showed a higher egg recovery rate and were then compared with de-ionized water for recovery of helminth eggs from hands. The highest recovery rate (95.6%) was achieved with a hand rinse performed with 7X 1%. Washing hands with de-ionized water resulted in an egg recovery rate of 82.7%. This washing method performed with a low concentration of detergent offers potential for quantitative investigation of contamination of hands with *Ascaris* eggs and of their role in human infection. Follow-up studies are needed that validate the hand washing method under field conditions, e.g. including people of different age, lower levels of contamination and various levels of hand cleanliness.

## Introduction

Ascariasis is an infection with the intestinal nematode *Ascaris lumbricoides* and it is estimated to infect over 800 million people worldwide [1]. Ascariasis is transmitted through the faecal-oral route; eggs are ingested following contact with contaminated hands, food, soil, or the deliberate act of eating contaminated soil. Infective *A. lumbricoides* eggs can survive, and remain infective for several months, or even for years in soil [2]. Eggs have been found on vegetables, especially in areas where excreta is used in agriculture [3], [4], on utensils, and even on banknotes [5].

In endemic areas infection with *A. lumbricoides* reaches maximum intensity and prevalence in children aged between 5 and 15 years [6] and is associated with impairments in growth and cognitive performance. Studies have shown that reducing the worm burden can lead to marked improvements in weight gain, school performance and nutritional status [7].

Considering the faecal-oral transmission route of ascariasis, improved hand hygiene should be an important control strategy, but it has been surprisingly under-researched. A recent systematic review concluded that access to, and use of, sanitation facilities could reduce the risk of *A. lumbricoides* and *Trichuris trichiura* infections by almost half [8]. However, evidence of an association between hand washing with soap and soil-transmitted helminths is inconclusive. Several studies have reported that the absence of soap within the household [9], absence of hand washing facilities in schools [10], or the low levels of hand washing with soap before eating or after defecation (self-reported) [11] are risk factors for ascariasis. However, none of these studies were able to disentangle these risk factors from poor socio-economic status or general hygiene, sanitation, or water availability variables. A systematic review that looked at promotion of hand washing with soap after defecation or before a meal as an intervention remained inconclusive, because one study showed a reduction of ascariasis while the other showed no impact [12].

The role of contaminated hands in the transmission of ascariasis is under-researched, with only a limited number of studies having investigated the presence, or the number of, eggs on hands. A study from Tajikistan reported that 34% of patients attending district health facilities were found to have *A. lumbricoides* eggs on their hands [13], while a study in Vietnam found that 13% of hand rinse samples collected from villagers of all ages in peri-urban Hanoi contained helminth eggs [14]. The first study [13] did not describe the method used to recover helminth eggs, while the second study [14] lacked details on methodology, and provided no information on the sensitivity of the method used.

The relative importance of hands in the transmission of *Ascaris* eggs may have been partly overlooked because validated methods are not available or published along with their performance. While methods to investigate bacteriological contamination of hands have been standardized and are routinely used [15], no such method is available to quantify helminth eggs concentrations on hands. The study presented here aimed at developing a method that would allow the quantification of *Ascaris* eggs - and possibly other helminth eggs - on hands, and to determine the recovery rate of the developed method.

## Materials and Methods

The method to assess the number of *Ascaris* eggs on hands was developed in the laboratory, with initial experiments carried out to optimize the egg recovery rate in each step of the method. These initial experiments included identification of two detergents (out of four that were evaluated) to be tested in the main hand washing experiment together with de-ionized water as a control. They also included validation of the egg counting technique, determination of the influence of pipette and falcon tube surfaces on egg recovery rate, and assessment of the recovery rate of eggs from the rinsing bags used. The main hand washing experiment then consisted of establishing and comparing the recovery rates of the two selected detergents and the de-ionized water on the hands of six volunteers.

### Recovery of helminth eggs

Eggs of *Ascaris suum* were recovered from fresh faeces of naturally infected pigs in Denmark. They were used as a model for *A. lumbricoides,* because they are virtually identical in morphology, size and surface properties [16]. *A. suum* eggs were isolated by a combination of sieving [17], [18] and flotation [19], [20]. A series of sieves of decreasing mesh size were used, starting with 500 µm, followed by 212 µm, 90 µm and 38 µm. Eggs recovered in the last sieve were concentrated by centrifugation at 253 *g* during 7 min and stored in demineralised water at 5°C with a concentration of approximately 11 eggs/μl until they were used, within 10 days. Normally eggs are stored in H_2_SO_4_ for prevention of fungal/bacterial growth in the egg solution, but this was not done in the current study due to concerns that the low pH might affect physico-chemical surface properties of the eggs. The same batch of eggs was used for both the initial experiments and the main hand washing contamination experiments.

### Initial experiments

#### Selection of rinsing solutions

Four different detergents were identified in the literature that were reported in previous studies to have been effective in recovering *Ascaris* eggs from contaminated vegetables, and in retrieving helminth eggs from sludge [21]. The detergents selected were safe to use on human skin at low concentration, reasonably cheap, and recommended for use for *Ascaris* eggs retrieval from sludge [22]. The detergents selected represented the groups of cationic (benzethonium chloride 0.1% and cetylpyridinium chloride CPC 0.1%), anionic (7X – quadrafos, glycol ether, and dioctyl sulfoccinate sodium salt 1%) and non-ionic (Tween80 - Polyethylene glycol sorbitan monooleate 0.1%) detergents. As ionic forces are likely to play a role in the adhesive properties of helminth eggs on various materials [23], at least one of each type was chosen for the initial experiments. For the main hand washing experiment, only the best performing two were selected, based on their *Ascaris* egg recovery rate in a standardized set-up with plastic bags (see details below). De-ionized water was used as a default rinsing solution.

#### Egg recovery rate by flotation techniques

A total of 1003 eggs (95% CI: 991-1015) in 90 µL of egg solution were added to ten 50 ml conical polypropylene centrifuge tubes (BD Falcon, San Jose CA, USA). The tubes were filled up to 50 ml with de-ionized water. Two different flotation techniques for counting helminth eggs were tested. In five of the tubes, the number of eggs was counted using a modified concentration McMaster technique [20], while the number of eggs in the remaining five tubes was determined using the FLOTAC method [24]. In brief, all 50 ml tubes were centrifuged at 253 *g* for 7 min and the supernatant was discarded, leaving approximately 1 ml of egg solution. The pellet was then re-suspended up to 5 ml in sugar-salt flotation solution (50 g glucose monohydrate/100 ml saturated NaCl solution yielding a density of 1.27 g/ml). For the counting of eggs, three McMaster slides (a total of 0.9 ml, 18% of the total sample) per tube were counted, while the total volume of 5 ml was used to perform a FLOTAC x 100 basic technique [24]. Non-coated plastic pipettes and Falcon tubes were used for all flotation steps.

#### Impact of pipette and falcon tube surfaces on helminth egg recovery

Published literature identified surface materials of laboratory consumables as potential modifiers of the adhesive properties of eggs [23]. Therefore, several combinations of pipettes and Falcon tubes - made of plastic or glass, coated or uncoated with organosilane (Rain-X Original Glass Treatment, Rain-X, USA) - were tested for egg recovery with the McMaster technique. For each combination, at least three replicates of 90 µl of egg solution, diluted in a 50 ml Falcon tube with de-ionized water were counted using three McMaster slides.

#### Egg recovery rates from rinsing bags

To test the efficacy of the McMaster technique to recover eggs in the bags used for the final hand washing experiment, polyethylene bags sized 17 cm by 25 cm with a grip seal were filled with 90 µl of egg solution and 40 ml of rinsing solution (de-ionized water or one of the four diluted detergents). The procedure is described below and was repeated in five bags for each rinsing solution. Each bag containing eggs and rinsing solution was massaged for 1 min (as done in the final hand washing experiment), and the content of the bag was transferred into a 50 ml Falcon tube by cutting one of the bag's lower corners. The sides and bottom of the bag were rinsed with de-ionized water from a squeeze bottle and the water was transferred into the same Falcon tube until filled to 50 ml. The eggs in the Falcon tube were then counted using the modified McMaster technique described in the paragraph above.

### Main hand washing experiment: Egg recovery rates from hands

For the main hand washing experiment six volunteers were found among the researchers in the Department of Veterinary Disease Biology at the University of Copenhagen to participate in this study. The volunteers were three women and three men aged 38 to 57 years. They were all trained in the hand rinsing method before the main hand washing experiment was initiated.

In order to limit the amount of time that each volunteer had to devote to the experiments, the number of rinsing solutions tested was limited to the two detergent solutions that showed the highest egg recovery rate in the initial experiments. The solutions tested were de-ionized water (control), 7X (Quadrafos, glycol ether and dioctyl sulfoccinate sodium salt), and benzethonium chloride. Three rinses of a hand pair contaminated with helminth eggs were done with each detergent and de-ionized water for each volunteer in a random order yielding a total of nine hand pair rinses per volunteer. The actual detergents used were unknown to the volunteers and to the researchers carrying out the experiments.

The full procedure for estimating the egg recovery rate from hands was as follows: the volunteer washed hands thoroughly for 1 min with laboratory liquid soap (Soft Care Wash H2, Diversey, USA) and rinsed them under flowing tap water for 1 min. Hands were then dried with single use paper towels. This was done to remove dirt and other substances in order to ‘standardize’ the surface of the hands before the experiment. A 90 µl volume of homogenized egg solution was then transferred to the palm of the volunteer's right hand with a micropipette, together with 160 µl of de-ionized water. The volunteer then spread the diluted egg solution over both hands by rubbing them together until dry without rubbing skin areas beyond the wrist line. Two polyethylene bags were each filled with 40 ml of one of the rinsing solutions (diluted detergent or de-ionized water) and each of the volunteer's hands was immediately introduced each in a separate bag. Rubber bands were then placed on the bags on the volunteer's wrists to avoid any spillage during the following massaging step. Volunteer's hands were massaged for 30 seconds by a researcher through the bag one after the other. The volunteer kept moving the hand not being massaged to keep the rinsing solution in movement. After opening the bags, the volunteer's hands were rinsed with approximately 5 ml of de-ionized water above the respective bags. Each bag content was then transferred into a 50 ml Falcon tube by cutting a lower corner of the bag, and sides/bottom of the bag were rinsed with approximately 5 ml of de-ionized water. The number of eggs in the Falcon tubes was counted using the modified McMaster method (as described above).

### Influence of hand washing before egg contamination and rinse with water

A last experiment was conducted to determine if the initial hand washing with laboratory soap had an influence on the egg recovery rate of de-ionized water in the main hand washing experiment due to laboratory soap residues on hand surfaces, and potential differences induced by natural skin condition. Therefore, hand rinses as described above in paragraph “Main hand washing experiment” were also performed without an initial hand washing with laboratory soap. In brief, a hand rinse with de-ionized water as described in paragraph “Main hand washing experiment” was performed on hands that had not been washed for at least one hour. After that, the volunteer washed his/her hands thoroughly with laboratory soap, 90 µl of egg solution was added to the hands as described above and another rinse with de-ionized water was performed and the eggs counted. This was repeated three times over three weeks on 5 volunteers, and once only on one volunteer.

### Data analysis

Data analysis was performed with STATA version 10.0 (Stata Corporation, College Station, USA). For the main hand washing experiment, egg counts for both hands (right and left hand) were pooled together for each volunteer, for each replicate, and examined for normality with a Shapiro-Wilk test for each rinsing solution. Considering that egg counts followed a normal distribution (p>0.05), t-test for equality of means and ANOVA were used to compare the performance of each rinsing solution, in terms of mean and variance across volunteers. An egg recovery rate was calculated by dividing the mean number of eggs recovered on hand pairs by the total number of eggs having been used for hand pair contamination. To determine if the hand washing or hand rinsing performed before hand contamination affected the outcome, egg recovery rates for each rinsing solution were stratified by previous detergent used, and compared using t-test.

### Ethical approval

The need for ethical approval to contaminate volunteers' hands with *A. suum* eggs extracted from fresh pig faeces was waived by the National Committee on Health Research Ethics in Denmark. However, we recruited the volunteers from the Department of Veterinary Disease Biology staff, University of Copenhagen, briefed them thoroughly on the study and offered to provide them with a free stool sample analysis and anthelminthic treatment after two months, at their own discretion. All the volunteers had years of experience, and in-depth knowledge on the risks of handling *A. suum* eggs. Volunteers were also informed that the detergents used were safe to use on skin at very low concentration, as reported in their respective Material Safety Data sheets.

We collected faeces from pigs from a private organic pig farm in Northern Zealand with the owner's permission, and the experiment did not involve any endangered or protected species. Faeces were collected from the rectum of pigs by digital palpation, with each pig only sampled once. No approval was obtained for collection of faecal samples from pigs as it is not required in Denmark. In cases of faecal sampling, approval from the experimental animal ethics committee is not required, according to Danish legislation (Lov om dyreforsøg/Law on Animal Experimentation, LBK No. 253 dated 8/03/2013, Ministry of Food, Agriculture and Fisheries of Denmark). The following is stated in this directive (Chapter 1, §2, second paragraph): “Animal experimentation includes any use of animals for scientific or educational purposes that supposedly will be associated with pain, suffering, anxiety or permanent damage equivalent to or stronger than introduction of a needle”. This “needle introduction” borderline is not violated in case of faecal sampling.

## Results

The mean number of *A. suum* eggs applied to the hand pair of an individual volunteer for each rinse was 1003 eggs (95% CI 991 - 1015), a dosage that was retained throughout the main hand washing experiment.

### Initial experiments to optimize egg recovery rate

Comparison of the mean egg recovery rate from five Falcon tubes processed by the two different flotation techniques found that the McMaster method used with non-coated pipettes had the highest recovery rate of 64.8% (95% CI 52.6-77.1). This egg recovery rate was higher than that which was obtained with organosilane-coated Falcon tubes, irrespective of the type of pipette used, while the FLOTAC basic technique had a mean recovery rate of 43.3% (95% CI 26.4–60.1) (Table 1). Based on these findings, the McMaster method was used in combination with non-coated Falcon tubes and pipettes in the following experiments.

**Table 1:** Helminth egg recovery obtained with different flotation techniques, tubes and pipettes and detergents.

	Method	Number of replicates	Mean number of eggs recovered in 90 µl (Standard deviation)	Mean egg recovery (%) (95% CI)
*Flotation techniques* a				
	Mod. McMaster (3 slides)	5 tubes	650.3 (90.3)	64.8 (52.6 – 77.1)
	FLOTAC basic method	5 tubes	433.8 (83.0)	43.3 (26.4 – 60.1)
*Tube/pipette material and coating*				
	Falcon tubes NC and glass pipettes NC^b^	5 tubes	585.6 (168.3)	58.4 (33.2 – 83.6)
	Falcon tubes NC and glass pipettes C^c^	5 tubes	571.2 (170.7)	57.0 (30.7 – 83.2)
	Falcon tubes C and glass pipettes C	3 tubes	335.2 (120.0)	33.4 (0 – 74.0)
	Falcon tubes C and plastic pipettes NC	3 tubes	374.1 (162.7)	37.3 (0 – 86.5)
	Falcon tubes C and glass pipettes NC	3 tubes	355.6 (188.2)	35.5 (0 – 95.4)
*Detergent*				
	Deionized water	5 bags	742.2 (56.2)	74.0 (67.3 – 80.7)
	7X 1%	5 bags	896.6 (228.1)	89.4 (67.1 – 100)
	Tween 80 0.1%	5 bags	586.6 (180.3)	58.5 (32.5 – 85.5)
	Benzethonium chloride 0.1%	5 bags	870 (129.2)	86.7 (73.7 – 99.8)
	Cetylpyridinium chloride 0.1%	5 bags	844.4 (178.1)	84.2 (65.7 – 100)

a Used with non coated Falcon tubes and plastic pipettes.

b NC  =  non-coated.

c C  =  coated with organosilane.

The results in Table 1 show that the highest egg recovery rate from polyethylene bags was found for detergent 7X (89.4%, 95% CI 67.1 – 100), followed by benzethonium chloride 0.1% (86.7%, 95% CI 73.7–99.8). The lowest rate was obtained with detergent Tween 80 (58.5%, 95% CI 32.5 – 85.5). We therefore selected detergents 7X and benzethonium chloride and de-ionized water (the latter as control) for the main hand washing experiment.

### Main experiment: recovery rates from volunteers' hands

The average estimated egg recovery rate for each rinsing solution was 95.6% (95% CI 89.6 – 100) for 7X, 88.2% (95% CI 79.2 – 97.2) for benzethonium chloride and 82.7% (95% CI 74.3 – 91.1) for de-ionized water (Table 2). On hands initially washed with soap, de-ionized water had the lowest egg recovery rate, which was statistically significantly lower than the rate obtained with detergent 7X. Detergent 7X also had the lowest variability in recovery rate across the six volunteers.

**Table 2:** Helminth egg recovery rates using different detergents for hand washing.

Detergent	Previous detergent^a^	Number of replicates	Mean number of eggs recovered for 90µl contamination dose (Standard deviation)	Mean egg recovery rate (%) (95% CI)
Deionized water				
	Laboratory soap	26	805.8 (221)	80.3 (69.8 – 90.9)
	7X and laboratory soap	5	871.1 (137.4)	86.9 (70.0–100)
	Benzethonium chloride and laboratory soap	3	968.5 (16)	96.6 (94.1 – 99.0)
	Any previous detergent	∑ 34	829.7 (207.1)	82.7 (74.3 – 91.1)
7X 1%				
	Laboratory soap	6	937 (179.9)	93.4 (78.0 – 100)
	7X and laboratory soap	5	980 (53.1)	97.7 (92.8 – 100)
	Benzethonium chloride and laboratory soap	7	962.7 (120.4)	96.0 (86.7 – 100)
	Any previous detergent	∑18	958.9 (124.9)	95.6 (89.6 – 100)
Benzethonium chloride 0.1%				
	Laboratory soap	5	800 (180.7)	79.8 (59.9 – 99.6)
	7X and laboratory soap	5	900 (194.9)	89.7 (70.7 – 100)
	Benzethonium chloride and laboratory soap	8	927.8 (155.4)	92.5 (80.9 – 100)
	Any previous detergent	∑18	884.6 (172.1)	88.2 (79.2 – 97.2)
Deionized water				
	None	16	724.6 (238.3)	72.2 (56.1 – 88.4)

a Previous detergent used on hands before the rinse was performed.

### Influence on egg recovery of previously used detergents

When stratifying pooled results by hand rinse solution, and the detergent used previously, results tend to show that a higher egg recovery rate was achieved when hands have been previously rinsed with either 7X or Benzethonium chloride, irrespective of the rinsing solution used in the experiment. However, the differences were not statistically significant (Table 2). Interestingly, rinses with de-ionized water on hands that were not previously washed with soap seemed to have a performance decreased by about 10% compared to a rinse with de-ionized water on hands previously washed with laboratory soap (single-sided t-test; p-value = 0.07).

## Discussion

This paper presents a method for the recovery of *Ascaris* eggs from hands, based on a hand rinse and modified McMaster eggs enumeration. When validated, the method showed a good recovery, ranging from 82.7% when de-ionized water was used for the hand rise to 95.6% when a diluted detergent was used.

To our knowledge, this is the first time that *Ascaris* egg recovery rates from hand rinses have been thoroughly tested and reported. The recovery rates reported for hands in this paper are similar to results obtained on vegetables (tomatoes) with the detergent 7X, which ranged between 96% and 100% [21]. However, our results for benzethonium chloride were marginally lower than those found on tomatoes in the same study.

The recovery rate in this study was optimized by testing several detergents, tube types and egg counting methods. In the limited literature on helminth egg adherence to surfaces, it appears that the physico-chemical forces determining egg adherence are complex, and most likely dependent on more factors than just the surface material that eggs might adhere to. Beyond ionic forces, adherence of eggs is likely to be modified by the pH of the solution containing the eggs, or by the age of the eggs (the surface properties of the eggs might change over time after shedding). In this study, plastic tubes and pipettes appeared more effective than glass tubes and pipettes. We also tested if coating the surfaces of pipettes and Falcon tubes with organosilane (usually used to coat car windshields as rain water repellent) would reduce the egg adherence by creating an inter-phase with favorable ionic and visco-elastic properties. However, egg adherence was not reduced with organosilane coating.

The two counting methods compared in this study - modified McMaster technique and FLOTAC - have been validated, particularly in the field of veterinary and human parasitology. When applied on animal or human stool samples analysis, both methods proved their efficacy [25]. However, our experiments show that on clean hand rinses, the modified McMaster technique had a much higher egg recovery rate. Given these findings, this technique was selected for the final experiment.

The use of plastic bags for hand washing is a simple modification of the ‘glove-juice’-method [15] developed for assessment of bacterial contamination of hands. Modification of the glove-juice method was necessary, because helminth eggs cannot undergo the same amplification (culturing) step before counting. Instead, in the present experiment, a concentration step (centrifuging) was needed. In order to achieve this, it was necessary to ensure that the entire rinsing solution that had been in contact with the hand was retrieved. Using a square plastic bag rather than a glove assisted in this step. Using 5 ml de-ionized water for rinsing each hand and the bag after the washing/massaging procedure with detergent was perhaps too small a volume for proper rinsing, but it was a necessary compromise. It enabled fitting both the detergent solution (40 ml), the rinsing water from one hand (5 ml), and the rinsing water from the bag (5 ml) into one 50 ml Falcon tube for one hand and minimized the number of handling steps, each of which can reduce the recovery rate.

The current knowledge on the influence of ionic forces in *Ascaris* eggs adherence does not explain the higher and less variable recovery rate achieved with diluted 7X, an anionic soap. 7X properties other than the electrical charge of its hydrophilic ends might actually account for its better performance in the reported experiments; for example, its pH, or its special design for laboratory glassware cleaning without leaving residues.

De-ionized water yielded fewer eggs from hands that were not previously washed than from hands that were previously washed with soap, suggesting that the initial hand washing step with laboratory soap either influenced the adherence of helminth eggs to the skin during hand contamination, or left surfactant residues that increased de-ionized water performance. Whatever the explanation, this emphasizes the need to further compare the performance of detergents on unwashed hands, in order to take into account possible interactions between the detergent and other factors (skin pH, oily or dry skin, presence of dirt) that may affect the method recovery rate and its variability across individuals and settings outside a controlled laboratory environment.

The hand rinsing method developed in this study can be performed in a field setting, potentially outdoors, whereas the enumeration of helminth eggs must be performed in a simple laboratory equipped with a centrifuge and a microscope. Washing naturally contaminated hands in the field using grip seal bags secured with rubber bands around their wrists does not require much explanation or training. The bags can then be sealed, preferably kept cool depending on the target helminth egg, and transported to the laboratory. In the laboratory, disposable non-sterile plastic pipettes and Falcon tubes are needed without any organosilane coating. Overall the method is quick, and easy to perform in the field and in areas with low-technology laboratories, i.e. many developing countries where most ascariasis burden occurs.

Based on this study, we suggest quantitative assessment of hand contamination with *Ascaris* eggs to be done with diluted 1% 7X detergent, with the method described, for an optimized recovery rate and less variation across individuals. However, we cannot be sure that both diluted cationic detergents and the de-ionized water will have comparable recovery rates in a field setting. The results reported here were obtained in a controlled laboratory setting, with a high contamination dose (1003 eggs for two hands) dispensed on clean hands, which will likely be different from hands found in fieldwork setting in terms both of cleanliness and the level of contamination with helminth eggs. We therefore recommend that the high egg recovery rates obtained with de-ionized water and diluted detergents are confirmed in experiments where hands are contaminated with a lower number of eggs. In fact, Hoa and colleagues [14] found only a single *Ascaris* egg on positive hands rinsed with diluted detergent, suggesting that naturally contaminated hands contain a much lower number of helminth eggs than the contamination dose used in the present study. We also recommend pursuing further validation in the laboratory with eggs of other common human helminthiases transmitted via the feco-oral route (*T. trichiura*, *Enterobius vermicularis* and *Taenia* spp) and on visibly dirty hands. Finally, a recent study in Dhaka, Bangladesh, showed that 51% of randomly selected dwellers near Dhaka University campus had *A. lumbricoides* eggs under their nails [26]. This indicates that future studies on retrieving helminth eggs from hands should determine any added value of examining under nail scrapings, especially in the presence of dirt.

The method presented here, if further validated, can be used to assess the effectiveness of complementing sanitation interventions with hand hygiene promotion to better prevent ascariasis infection. It will enable us to quantify the range of hand contamination with *Ascaris* eggs found in high, medium and low transmission settings and measure the risk factors for high hand contamination with helminth eggs. Further studies including this method among others could investigate the relative importance of the main ascariasis infection routes (pica, contaminated raw food and hands).
